# Gut Microbiota Conversion of Dietary Ellagic Acid into Bioactive Phytoceutical Urolithin A Inhibits Heme Peroxidases

**DOI:** 10.1371/journal.pone.0156811

**Published:** 2016-06-02

**Authors:** Piu Saha, Beng San Yeoh, Rajbir Singh, Bhargavi Chandrasekar, Praveen Kumar Vemula, Bodduluri Haribabu, Matam Vijay-Kumar, Venkatakrishna R. Jala

**Affiliations:** 1 Department of Nutritional Sciences, The Pennsylvania State University, University Park, Pennsylvania, United States of America; 2 Department of Medicine, The Pennsylvania State University Medical Center, Hershey, Pennsylvania, United States of America; 3 Department of Microbiology and Immunology, James Graham Brown Cancer Center, University of Louisville, Louisville, Kentucky, United States of America; 4 Institute for Stem Cell Biology and Regenerative Medicine (inStem), UAS-GKVK Campus, Bellary Road, Bangalore, Karnataka, India; 5 Ramalingaswami ReEntry Fellow, Dept. of Biotechnology, Govt. of India; University of Catania, ITALY

## Abstract

Numerous studies signify that diets rich in phytochemicals offer many beneficial functions specifically during pathologic conditions, yet their effects are often not uniform due to inter-individual variation. The host indigenous gut microbiota and their modifications of dietary phytochemicals have emerged as factors that greatly influence the efficacy of phytoceutical-based intervention. Here, we investigated the biological activities of one such active microbial metabolite, Urolithin A (UA or 3,8-dihydroxybenzo[c]chromen-6-one), which is derived from the ellagic acid (EA). Our study demonstrates that UA potently inhibits heme peroxidases i.e. myeloperoxidase (MPO) and lactoperoxidase (LPO) when compared to the parent compound EA. In addition, chrome azurol S (CAS) assay suggests that EA, but not UA, is capable of binding to Fe^3+^, due to its catechol-like structure, although its modest heme peroxidase inhibitory activity is abrogated upon Fe^3+^-binding. Interestingly, UA-mediated MPO and LPO inhibition can be prevented by innate immune protein human NGAL or its murine ortholog lipocalin 2 (Lcn2), implying the complex nature of host innate immunity-microbiota interactions. Spectral analysis indicates that UA inhibits heme peroxidase-catalyzed reaction by reverting the peroxidase back to its inactive native state. In support of these *in vitro* results, UA significantly reduced phorbol myristate acetate (PMA)-induced superoxide generation in neutrophils, however, EA failed to block the superoxide generation. Treatment with UA significantly reduced PMA-induced mouse ear edema and MPO activity compared to EA treated mice. Collectively, our results demonstrate that microbiota-mediated conversion of EA to UA is advantageous to both host and microbiota *i*.*e*. UA-mediated inhibition of pro-oxidant enzymes reduce tissue inflammation, mitigate non-specific killing of gut bacteria, and abrogate iron-binding property of EA, thus providing a competitive edge to the microbiota in acquiring limiting nutrient iron and thrive in the gut.

## Introduction

Gut microbiota is considered as hidden metabolic ‘organ within an organ’ because of its immense effect on health by influencing the host nutrient acquisition, metabolism, physiology, and immune functions [1–4]. The human gastrointestinal tract (GIT) harbours complex microbial community as high as up to ~10^14^ microorganisms. One of the major functions of gut microbiota is to metabolize dietary components to a spectrum of metabolites. Several of these microbial-derived metabolites aid in developing and controlling immune system not only in the gut but also distant organs. The balance between microbial controlled pro and anti-inflammatory activities is critical to maintain gut homeostasis and regulate gut inflammation and colon carcinogenesis [5–10]. Several external factors such as infection, diet and usage of antibiotics are known to disrupt the dynamics of microbial communities in the intestine and the host immune system [8, 11–17]. Efforts are currently underway to define complex microbiota-host interactions (good *vs* bad; cause *vs* consequence) using animal models in germ-free conditions and antibiotic-mediated microbiota ablation [15, 18–21]. However, given the complexity of gut microbiota and their metabolites, it is difficult to predict which specific bacterial pool is responsible for beneficial or opportunistic/pathogenic effects solely based on microbiota/metagenomics analysis. Further, the influence of microbial metabolites in immune-modulatory function adds another layer of complexity.

The health benefits rendered by consumption of several natural plant products (e.g., pomegranates, walnuts and berries) have been associated with their high levels of health promoting polyphenolic compounds, specifically ellagitannins and EA [22–24]. These compounds display protective effects against chronic metabolic disorders both in preclinical and clinical studies [24–30]. The beneficial effects of EA are associated with multi-target action that involve anti-inflammatory, anti-oxidant and anti-carcinogenic effects [24, 31]. However, the intestinal absorption (bioavailability) of ellagitannins and EA is limited [32–34]. Recently, Gonzalez-Sarrias et al demonstrated the limits for EA bioavailability in healthy volunteers after consumption of pomegranate extracts [35]. These studies reported that EA bioavailability is not as low as previously described. However, pharmacokinetics suggested that there is high inter-individual variation of EA bioavailability Cmax ranging from 12 to 360 nm [35]. It has been suggested that potential health benefits rendered by these compounds *in vivo* are due to gut microbiota-mediated conversion into metabolites called urolithins [31, 36]. Urolithins [36] are microbial metabolites derived from EA or ellagitannins by commensal bacteria [37] and are dibenzopyran-6-one derivatives with different hydroxyl group substitutions. If appropriate gut microbiota is present in healthy individuals, urolithins can reach up to micromolar concentrations in the plasma of humans [33] as well as in different target tissues of animal models [38–40] without any toxicity. Among urolithins, Urolithin A (UA; 3,8-dihydroxybenzo[c]chromen-6-one) has been shown to influence the microbiota composition in rat models [38], but the significance of these changes remain to be established. Basic pharmacodynamics studies of UA have been established in both humans and mice models (reviewed by Epsin JC et al [31]). These studies highlight that the concentration of UA reaches up to micromolar (μM) without displaying any toxic effects *in vivo*. For instance, upon consumption of pomegranate juice by humans, peak plasma levels of UA could reach from 14 to 40 μM but with a large individual-variations [33, 41]. Such variation could be due to that the bacteria responsible for urolithins production may or may not be present in all individuals. Recently, Selma et al identified the mono cultured bacteria (*Gordonibacter urolithinfaciens* and *Gordonibacter pamelaeae* DSM 19378^T^) that are responsible for metabolizing the EA to produce luteic acid, UroM-5, UroM-6 and UroC [37]. However, these cultured bacteria are incapable of producing the downstream products, UroA (UA) and UroB. Thus, several efforts are now underway to determine the bacterial phyla or group of bacteria responsible for production of UroA and UroB, which can potentially serve as probiotics.

Myeloperoxidase (MPO) is a 150-kDa protein belonging to the heme peroxidase superfamily that includes eosinophil peroxidase, lactoperoxidase (LPO), thyroid peroxidase, and prostaglandin H synthase [42]. It is predominantly expressed in neutrophils and mediates the generation of hypochlorus acid from hydrogen peroxide (H_2_O_2_) and chloride anion (Cl^-^) during respiratory burst of neutrophils. Heme group is critical to catalyze these actions [43]. MPO-produced hypochlorous acids are cytotoxic and thus are utilized in killing bacteria by neutrophils as part the host innate defense mechanism [44, 45]. However, they also contribute to tissue damage and perpetuation of inflammatory disorders including inflammatory bowel disease (IBD) [46–49]. Accordingly, there is significant interest in developing MPO inhibitors as potential strategy to mitigate the adverse effects of MPO during inflammation. To date, several naturally occurring polyphenolic compounds have been reported to act as inhibitors of MPO such as harmala alkaloids [50] and epigallocatechin-3-gallate (EGCG) [51].

The aim of the study is to determine the potential beneficial anti-inflammatory and peroxidase inhibitory activities of UA compared to EA in *in vitro* and *in vivo* models. Here, we examined the effects of EA and UA on activities of MPO/LPO, interdependence of Ferric iron and host neutrophil gelatinase-associated lipocalin (human NGAL) and its murine ortholog lipocalin 2 (Lcn2). We also tested the efficacy of EA and UA in phorbol 12-myristate 13-acetate (PMA)-induced ear edema model. Altogether, our findings provide insights into interplay between microbiota-derived phytometabolites and host NGAL/Lcn2 in the complex regulation of heme peroxidases.

## Materials and Methods

### Materials

Mouse recombinant (rec)-Lcn2 was obtained from Cell Signalling and human rec-NGAL was acquired from R&D Systems; both of which are free from endotoxin, siderophore, and iron. Human MPO was procured from R&D Systems (Minneapolis, MN). EGCG, PMA, ferric chloride, PIPES, agar, and H_2_O_2_ were procured from Sigma-Aldrich (St. Louis, MO). Bovine milk LPO was purchased from Worthington Biochemical Corp (Lakewood, NJ). Chrome Azurol S was purchased from Acros Organics. Guaiacol (2-methoxyphenol) was obtained from Alfa Aesar (Ward Hill, MA).

### Synthesis of UA

In a 500 ml round bottom flask, a solution of 2-bromo-5-methoxy benzoic acid (10gm, 0.043 moles) and AlCl_3_ (17.26 gm, 0.129 moles) in chlorobenzene (230 ml) was added and the reaction was refluxed for 16 hrs at 131°C. The product obtained was extracted using ethylacetate and concentrated using rotary evaporator. In a two necked round bottom flask, the demethylated product (2-bromo-5-hydroxy benzoic acid) obtained in reaction 1 (8.7 gms, 0.04 moles) along with resorcinol (4.414 gm, 0.04 moles), sodium hydroxide (4 gm, 0.10 moles) was mixed with water (45.5 ml) and reaction was refluxed for 2 hrs at 100°C. To the above mixture, 10%w/v CuSO_4_ (17 ml) was added and refluxed for another 2 hrs. Crude product was purified by column chromatography. The desired compound was eluted with a solvent mixture of hexane: ethyacetate (50:50) followed by recrystallization in ethylacetate to ensure high purity. The purified UA (1 g) was concentrated and characterized by TLC, NMR spectroscopy, mass spectroscopy and HPLC (S1 Scheme).

### MPO and LPO *in vitro* Assay

Stock solutions of EA (10 mM) and UA (100 mM) were prepared in Dimethyl sulfoxide (DMSO, sigma) and subsequent dilutions were prepared in PBS. MPO and LPO assays are based on the principle that peroxidase oxidizes guaiacol (2-methoxyphenol) in the presence of H_2_O_2_ to a chromophore 3,3’-dimethoxy-4,4’ biphenylquinone [52]. The change in absorbance at 470 nm was measured per minute intervals over a period of 10 min. The reactions were executed at pH 6.0 and 25°C in 96-well plates (Corning, NY) in triplicate using appropriate vehicles and buffer controls throughout the study. One unit of MPO activity was defined as the amount that increases absorbance at 470 nm by one per minute at 25°C, calculated from the initial rate of reaction with the use of guaiacol as the substrate. Bovine milk LPO and Human MPO were reconstituted with 0.1 mol/L potassium phosphate buffer, pH 6.0 and stored at -80°C. MPO (12.5 μg/ml, final reaction concentration) or LPO (25 μg/ml, optimum concentration) were pre-incubated with different concentrations of EA and UA (0–100 μM) with or without mLcn2/hu NGAL or iron (FeCl_3_) for 5 min at room temperature in 20 μl reaction mixture. The reaction was initiated on the addition of 30 μL of H_2_O_2_ (6.7x10^-3^%) and 50 μL of 100 mmol/L guaiacol (prepared in 0.1 mol/L potassium phosphate buffer). The change in absorbance at 470 nm was recorded per minute intervals over a period of 10 min.

### CAS agar Assay

Chrome Azurol S (CAS) agar plates were prepared according to procedure described by Schwyn and Neilands [53]. The principle of CAS assay is CAS remains blue in color when complexed with iron but turns into orange color halo when iron is chelated by iron chelators. In CAS agar plate, formation of orange halos indicates the iron chelation properties of the given compound. Briefly, 2 μL of EA or UA mixed with or without FeCl_3_ at indicated concentrations were placed on CAS plate and monitored for orange-color halo formation. The intensity of halo formation is directly proportional to the concentration of chelated iron. EGCG (Sigma-Aldrich) was used as a positive control [51].

### CAS liquid assay

CAS reagent was prepared according to the procedure described by Payne in 1994 [54]. Briefly, EA or UA (0–100 μM, 100 μl) and EGCG (positive control) were incubated with CAS reagent (100 μl) for 20 min in a 96 well plate at room temperature and absorbance was monitored at 630 nm. The percent iron chelation was calculated from the absorbance difference against blank (vehicle).

### Spectral Analysis

To perform spectral analysis, LPO (1.0 mg/ml) was reconstituted in 500 μl of 0.1 mol/l phosphate buffer (pH 6.0) and basal spectra were recorded at 412 nm for freshly reconstituted LPO. The reaction was initiated when 30 μM H_2_O_2_ was added to LPO, followed by addition of either EA (50 μM), UA (50 μM) or vehicle. Spectra were recorded throughout the reaction at 300 to 500 nm every 3 seconds using CARY50BIO UV-Visible Spectrophotometer. Each spectrum represents an average of six scans. All reaction concentrations above represent final system concentrations.

### Isolation of bone marrow derived neutrophils (BMDNs)

Murine bone marrow cells from WT C57BL/6 mice were isolated and neutrophils were separated by density gradient centrifugation as described by Swamydas and Lionakis [55]. BMDNs were prepared using Histopaque gradient (Sigma) method [55] and obtained >95% pure and >99% viable neutrophils, which was confirmed with flow cytometry.

### Nitroblue tetrazolium (NBT) reduction assay

BMDN cells (2x10^5^ cells/well) were seeded on a 96 well in triplicates with a working concentration of NBT (1 mg/mL) with and without PMA (50 nM) in presence of EA or UA (50 μM) and then incubated at 37°C for 3 hrs. Images of cells were taken after incubation and a minimum of 100 cells were observed in each well for NBT-positive cells (%). The cells were fixed using 200 proof ethanol at -20°C for 15 minutes followed by two washes in 70% ethanol. Wells were allowed to dry and 140 μL of KOH (2M) was added to lyse all cells followed by 160 μL of DMSO to solubilize the formazan. After homogenization of the contents in the wells, the absorbance was read at 620 nm [56].

### PMA-induced ear edema

All the animal protocols were approved by IACUC at University of Louisville, Louisville, KY. C57BL/6 mice (6–8 weeks old) were purchased from Jackson laboratories. Acute PMA-induced ear edema was performed according to method described previously [57]. Briefly, mice were divided into 4 groups (n = 5) *viz*. vehicle (10% glucose), UA (40 mg/kg body weight), EA (40 mg/kg body weight) and indomethacin (10 mg/kg body weight); indicated treatments were delivered orally (100 μl) 2 hrs before the application of PMA. PMA (4 μg/ear in 20 μL acetone) was applied to inner surface on right ear of each mouse while left ear of each mouse received vehicle (20 μL acetone) on inner surface. At six hours post-application of PMA, animals were sacrificed by cervical dislocation and 6 mm diameter disc from each ear was removed using metal punch. The tissues were weighed and ear edema weight was calculated by subtracting the weight of the left ear from the right ear (treatment). Percent inhibition was calculated and expressed as decrease in weight with respect to control. Tissue samples obtained from each mouse were also assessed for MPO activity using Bradley et al method [58]. Briefly, tissue (6 mm) was homogenised in 50 mM phosphate buffer containing 0.5% hexadecyl trimethylammonium bromide using polytron homogenizer (Ultra-turrax T8). Samples were centrifuged after three freeze-thaw cycles (-80°C and 37°C) at 5000 rpm for 20 min at 4°C. Supernatant was used to measure MPO activity. Briefly, 10 μL of sample was mixed with 190 μL of 50 mM phosphate buffer (pH 6.0) containing 0.167 mg/mL *O*-dianisidine and 0.005% H_2_O_2_. The change in absorbance was measured at 460 nm using Synergy HT spectrophotometer (Biotek Instruments Inc.) MPO activity results were represented as units per 6 mm of tissue. One unit of MPO activity was defined as that degradation of 1 mM peroxide per minute at 25°C.

### Statistical Analysis

All experimental results were reproduced in at least three independent experiments performed in triplicates. All values in the results are expressed as mean ± SEM. The significance of difference between different groups was determined by unpaired Student t-test in case of two groups. P-value less than 0.05 were considered statistically significant. GraphPad Prism version 6.0 (GraphPad Software Inc., La Jolla, CA) was used to calculate statistical significance.

## Results

### Synthesis and characterization of Urolithin A (UA)

UA has been synthesized from 2-bromo-5-methoxy benzoic acid in two steps as described in Methods section. The purified UA was obtained as slight yellow color solid, which was characterized using NMR spectroscopy, mass spectroscopy and HPLC (S1 Scheme, S1 and S2 Figs). High performance liquid chromatography (HPLC, S3 Fig): HPLC was performed to quantify the amount of Urolithin-A. The mobile phase consisted of 50% of Acetonitrile and 50% of Water:Formic acid (99:1).The solvent used to dissolve the compound was tetrahydrofuran with an injection volume of 20 μl. The flow rate through the CAPCELL PAK C18 Column (4.6mm× 250mm, SHISEIDO) was 1 ml/min for 10 minutes with post run time of 50 seconds and column temperature being 30.0°C. The UV detection wavelength for the drug was 280nm. Urolithin-A was shown RT of 4.1 min (S3 Fig).

### UA inhibits MPO and LPO in dose and time dependent manner

**Fig 1:**
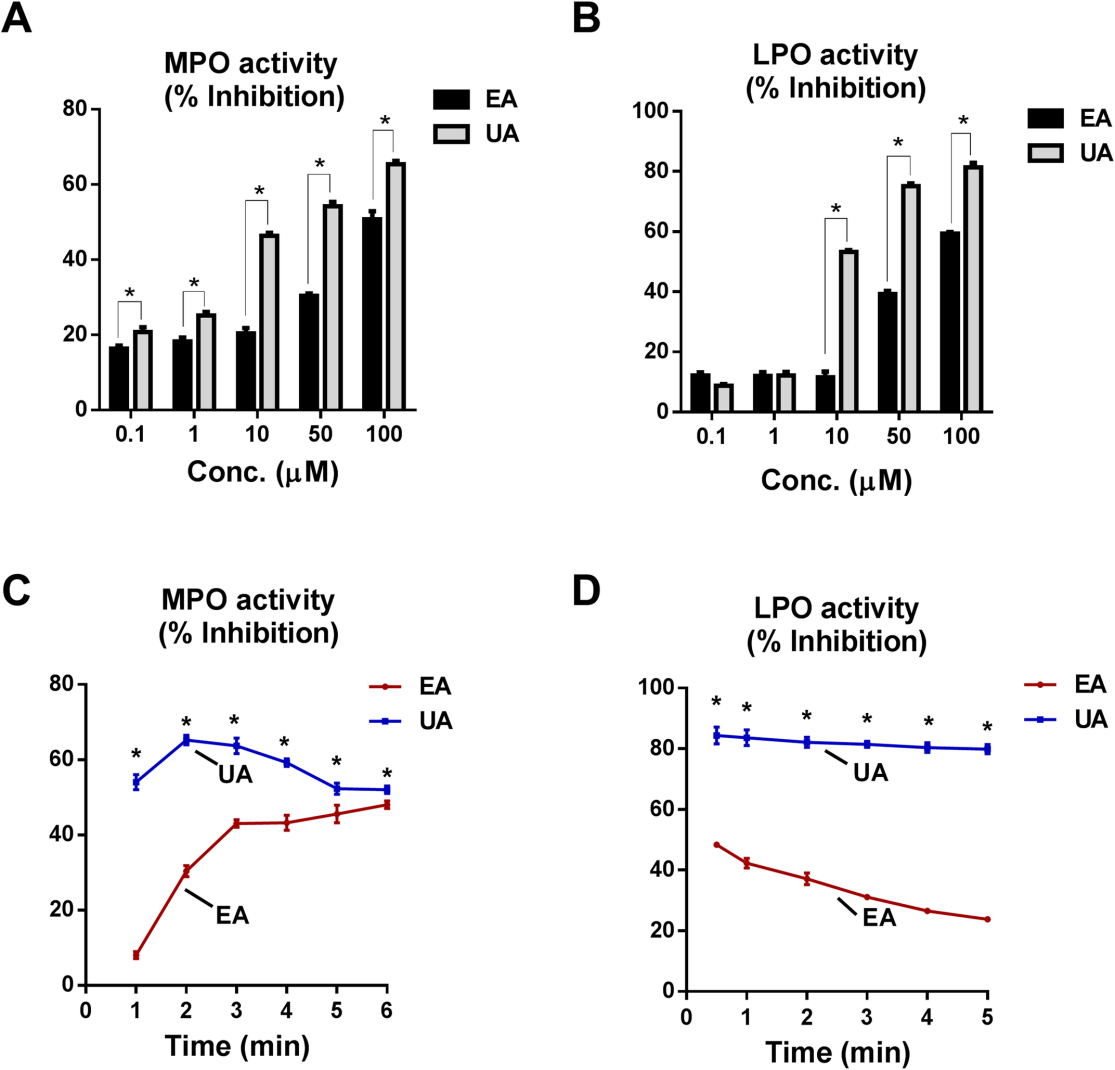
EA and UA inhibit MPO and LPO activity in dose dependent manner: EA or UA (0–100 μM) was pre-incubated at the indicated final concentrations with MPO (12.5 μg/ml) or LPO (25 μg/ml) for 5 min in 20 μl reaction volume before addition of 30 μl of H_2_O_2_ (6.7x10^-3^%) and 50 μl of 100 mmol/L guaiacol. Bar graph represent EA and UA mediated dose-dependent inhibition of MPO (A) and LPO (B) activity. Line graph represents EA (50 μM) and UA (50 μM) mediated inhibition of MPO (C) and LPO (D) activity with time. Assays were carried out in 96-well plates in triplicates with appropriate vehicles/buffers. Results are representative of three independent experiments and are expressed as mean ± SEM. *p < 0.05.

EA and their microbial metabolites have been shown to have anti-inflammatory and anti-oxidative activities [31], although their underlying molecular mechanisms are not completely understood. Here, we investigated the effects of parent compound, EA and its microbial metabolite, UA on the pro-oxidant activities of MPO and LPO. As shown in Fig 1, EA and UA significantly reduced the MPO and LPO activities in both dose (Fig 1A and 1B) and time dependent manner (Fig 1C and 1D). Most importantly, UA at 10 μM concentration potently inhibited MPO activity, whereas EA show an approximately two-fold reduced inhibitory activity at this concentration. Our analysis suggested that UA is 10-times more effective than EA, given that 10 μM of UA could inhibit MPO activity by 60% whereas 100 μM EA was required to achieve equivalent level of inhibition. Similarly, UA also inhibited the activity of LPO, which is another heme-containing peroxidase (Fig 1B and 1D). It is intriguing that UA potently inhibited MPO activity within a minute after addition to the reaction mixture, whereas EA-mediated inhibition was relatively slower that gradually reaches its optimal inhibitory effect at 6 min after initiation of MPO activity (Fig 1C). The inhibitory potency of UA was also observed to much stronger towards LPO than MPO (Fig 1D).

### UA fails to chelate ferric iron

**Fig 2:**
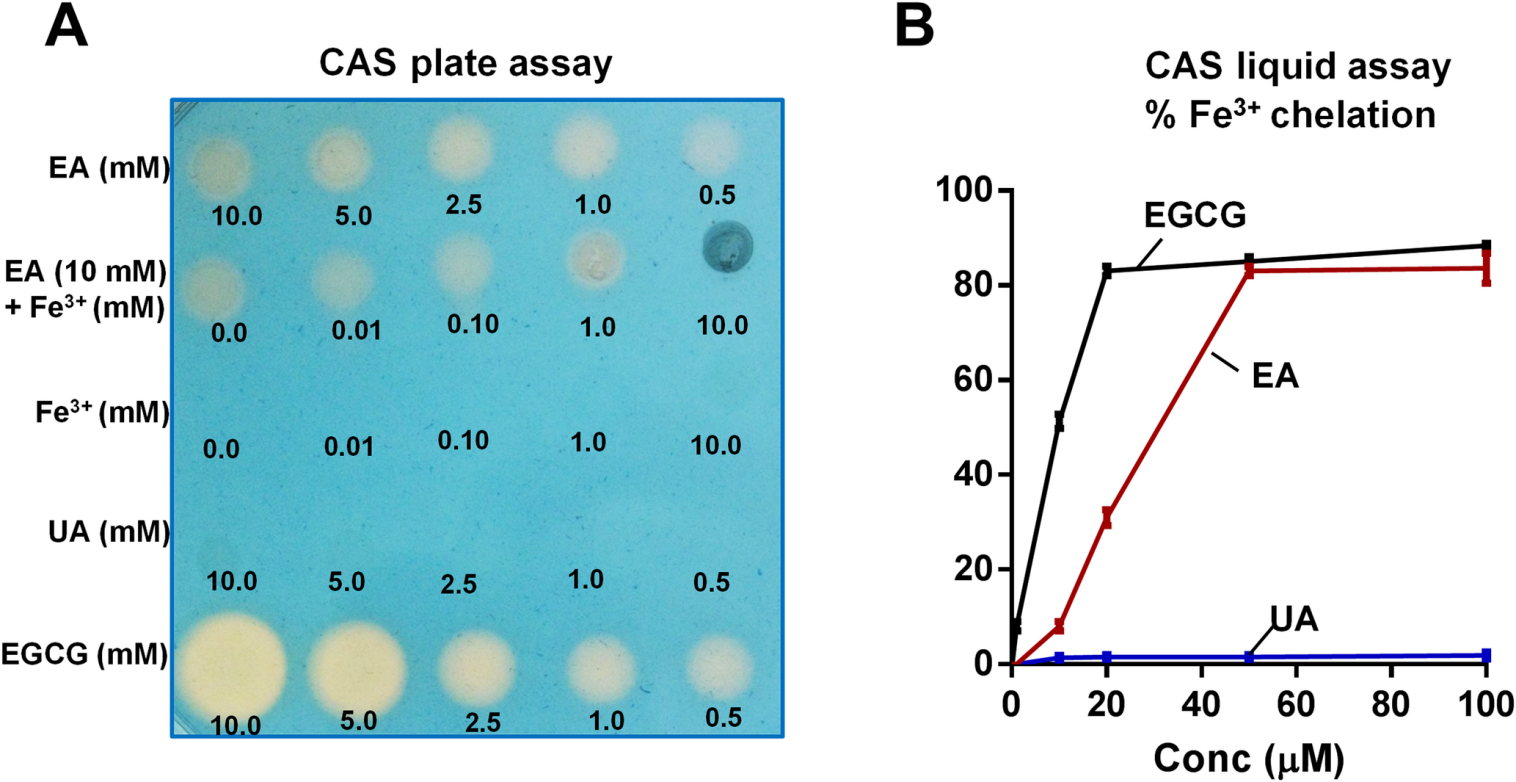
EA, but not UA, can chelate iron. (A) EA or UA (1–10 mM) were pre-incubated with or without Fe^3**+**^ at indicated molar ratio and then 2 μl of the reaction mixture was applied on Chrome azurol S (CAS) plate. Formation of orange halo indicated chelation of iron in the CAS plate. EGCG and FeCl_3_·6H_2_O were used as positive and negative controls, respectively. In the liquid CAS assay, EA or UA (0–100 μM, 100 μl) and EGCG (positive control) was incubated with CAS reagent (100 μl) for 20 min and absorbance was monitored at 630 nm; % chelation was calculated against blank. (B) Line graphs represent the % chelation of Fe^3+^ by EA, UA and EGCG. Results are expressed as mean ± SEM. Assays were performed in a 96-well plate in triplicates and are representative of three independent experiments. *p < 0.05.

Since many plant-derived polyphenols (i.e. epigallocatechin-3-gallate or EGCG) are known to bind iron [59], therefore we next asked whether iron-binding could affect the peroxidase inhibitory activity of EA and UA. Accordingly, we employed the chrome azurol S (CAS) assay to test for the ability of EA and UA in chelating iron, using EGCG as the positive control. As shown Fig 2A, CAS agar plate assay demonstrated that EA and EGCG are both capable of binding iron, whereas UA failed to chelate iron. This could be attributed the presence of catechol group (2-OH groups one phenyl group next to each other) on EA responsible for iron chelation, which is absent in UA. We also confirmed these results using CAS liquid assays and represented as percent Fe^3+^ chelation (Fig 2B). Our spectral analysis of UA further confirmed that it is completely free from iron (data not shown).

### Ferric iron modestly counter-regulates the peroxidase inhibitory activity of UA, but completely abrogates the inhibitory activity of EA

**Fig 3:**
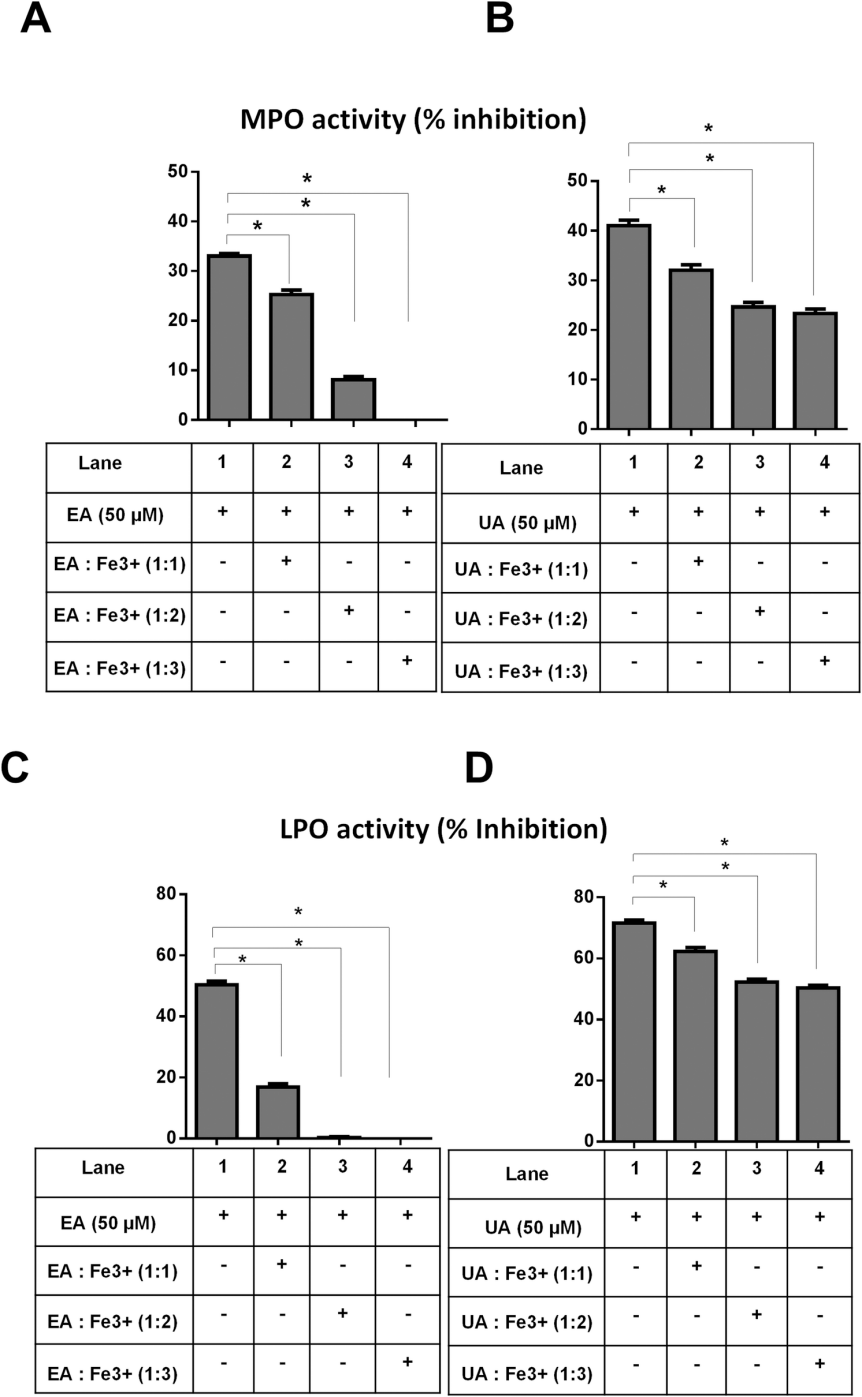
Fe^3+^bound EA, but not UA, failed to inhibit heme peroxidase activity: UA (10 μM) or EA (10 μM) and indicated molar ratio of FeCl_3_·6H_2_O were pre-incubated at room temperature with either MPO (12.5 μg/ml) or LPO (25 μg/ml) for 5 min before the assay was initiated. Bar graphs represent inhibition of MPO activity (A) EA+ Fe^3+^ and (B) UA+ Fe^3+^ at indicated molar ratio. Bar graph represents inhibition of LPO activity by (C) EA and (D) UA in presence of Fe^3+^ at indicated molar ratio. Results are expressed as mean ± SEM. Assays were performed in a 96-well plate in triplicates and are representative of three independent experiments. *p < 0.05.

We previously reported that iron-free, but not iron-saturated EGCG could potently inhibit the activity of heme peroxidases [51]. In a similar fashion, we observed that adding EA and ferric iron at molar ratio of 1:3 completely abrogated the ability of EA to inhibit the activity of MPO (Fig 3A). However, the increased molar concentration of UA:Fe3^+^ from 1:0 to 1:1 only modestly mitigated UA inhibitory activity, whereas the increment from 1:1 to 1:3 did not result in further significant reduction of MPO activity (Fig 3B). Similar results were observed, when we repeated the experiment on LPO activity (Fig 3C and 3D). The greater susceptibility of EA than UA inhibitory activity to be abrogated by Fe^3+^ may be due to EA’s ability to chelate iron, whereas UA does not bind iron. These findings implicate a unique advantage of UA over EA as a heme peroxidase inhibitor that could withstand Fe^3+^ counter-regulatory effects.

### Innate immune protein lipocalin 2 mitigates the inhibitory heme peroxidase activity of UA

**Fig 4:**
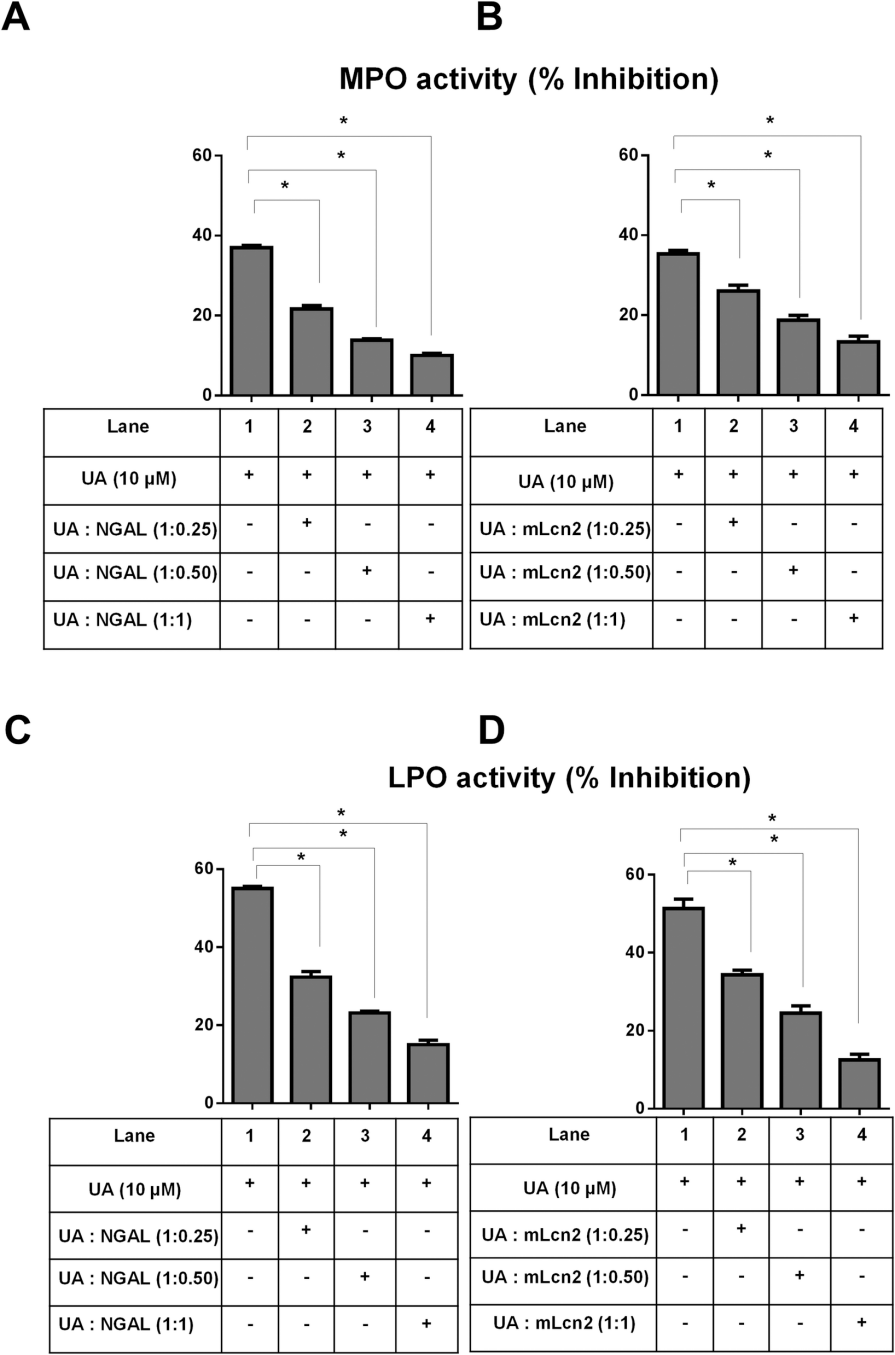
Human NGAL and its murine orthologue Lcn2 inhibit UA-mediated MPO and LPO inhibition. UA (10 μM) and indicated molar ratio of NGAL/mLcn2 were pre-incubated at room temperature with either 12.5 μg/ml MPO or 25 μg/ml LPO for 5 min before the assay was initiated. Bar graphs represent inhibition of MPO activity by UA in the presence of (A) NGAL and (B) mLcn2 at indicated molar ratio. Bar graph represents inhibition of LPO activity by UA in presence of (C) NGAL and (D) mLcn2. Results are expressed as mean ± SEM. Assays were performed in a 96-well plate in triplicates and are representative of three independent experiments. *p < 0.05.

The multifaceted innate immune protein murine Lipocalin 2 (Lcn2) and its human orthologue NGAL have been demonstrated to protect MPO activity from being inactivated by bacterial siderophore enterobactin [60] and EGCG [51]. Lcn2 are expressed in neutrophils and play an important role in limiting bacterial growth by sequestering iron-containing siderophores. Expression of Lcn2 as well as MPO is significantly increased during intestinal inflammation [61, 62]. Herein, we examined whether presence of murine Lcn2 and human NGAL could have any influence on UA-mediated inhibition of MPO and LPO activity. As shown in Fig 4A and 4C, NGAL significantly reduced UA-mediated inhibitory activity of both MPO and LPO in a dose dependent manner. The addition of NGAL to 10 μM UA at molar ratio of 1:1 reduced the percent UA-mediated MPO inhibition from ~40% to ~10%, which translates to approximately 3.5-fold loss of UA bioactivity. We observed similar mitigation of UA inhibitory effect on LPO as well (from ~60% to ~17% inhibition) when NGAL was added to UA at 1:1 molar ratio. Similar to NGAL, mouse orthologue, Lcn2 also significantly mitigated UA heme peroxidase inhibitory activity (Fig 4B and 4D). However, we were not able to perform similar analysis on EA due to technical limitation *i*.*e*. 100 μM of EA are needed to achieve significant MPO/LPO inhibition, but NGAL/Lcn2 are not readily supplied at amount feasible to achieve 1:1 ratio with 100 μM of EA. Our experiment with 10 μM EA with NGAL/Lcn2 at 1:1 ratio failed mitigate the bioactivity of EA (data not shown) probably because EA bioactivity was already low (~15–20%) and could not be mitigated further.

### UA and EA inhibit heme peroxidase by inactivating peroxidase-catalysed reaction

**Fig 5:**
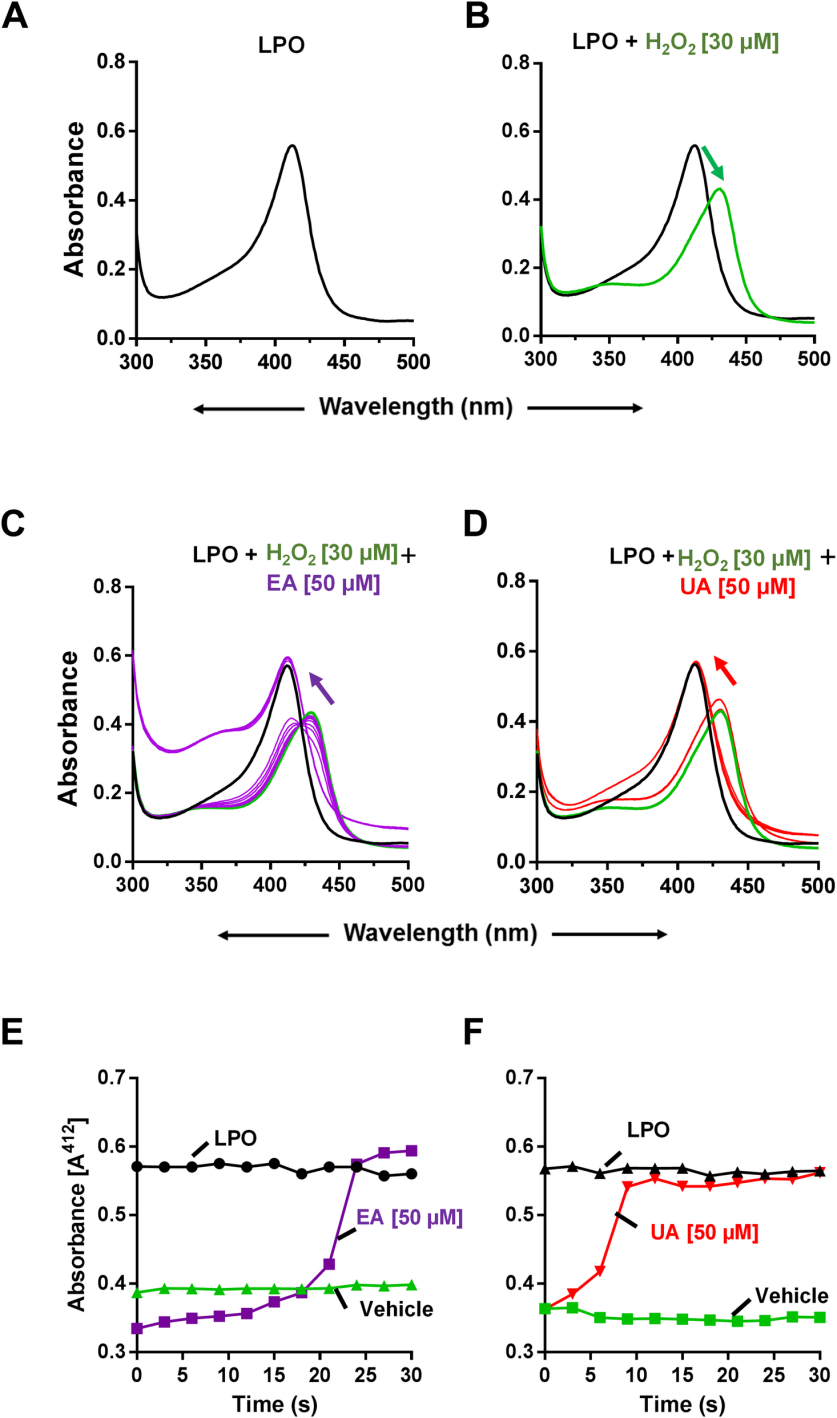
EA and UA avert the heme peroxidase-catalysed reaction. To perform spectral analysis, LPO (1 mg/ml, in 0.1M phosphate buffer) + H_2_O_2_ (30 μM) was incubated with either 50 μM of EA or UA. Spectra were recorded at 300 to 500 nm at every 3 seconds. Each spectrum represents an average of six scans. Image represents (A) LPO alone, (B) LPO + H_2_O_2_, (C) LPO + H_2_O_2_+ EA and (D) LPO + H_2_O_2_+ UA_._ Arrows designate the direction of spectral changes over time on the initiation of the reaction. Kinetics of spectral changes were recorded upon addition of (E) EA or (F) UA or vehicle to LPO-H_2_O_2_ reaction. Results are representative of three independent experiments.

LPO displays maximum absorbance (λ_max_) at 412 nm due to the presence of heme moiety group in the enzyme (Fig 5A). To determine whether UA or EA interacts with heme group of LPO, we recorded the absorbance spectra of LPO in the presence of UA or EA. However, the addition of UA or EA did not result in any changes in the spectra of LPO (data not shown), indicating that mechanism of inhibition by UA/EA does not involve heme iron-binding. Next, we tested whether UA/EA have effect on LPO-H_2_O_2_ spectral properties. We knew that, upon the addition of H_2_O_2_ to LPO, the heme iron (Fe^3+^) of LPO would interacts with H_2_O_2_ and produce redox intermediate called oxoiron with λ_max_ at 430 nm (Fig 5B**)**. The addition of EA (Fig 5C) or UA (Fig 5D) reverted the LPO-H_2_O_2_ spectra (λ_max_) from 430 nm back to 412 nm. These observations indicated that these compounds reverted the redox reaction and restored the native Fe^3+^ state of heme of LPO. Intriguingly, the UA-mediated spectral shift was noticeably more rapid and faster than the slower spectral shift mediated by EA (Fig 5E and 5F).

### UA inhibits PMA-induced generation of reactive oxygen species (ROS) in neutrophils

**Fig 6:**
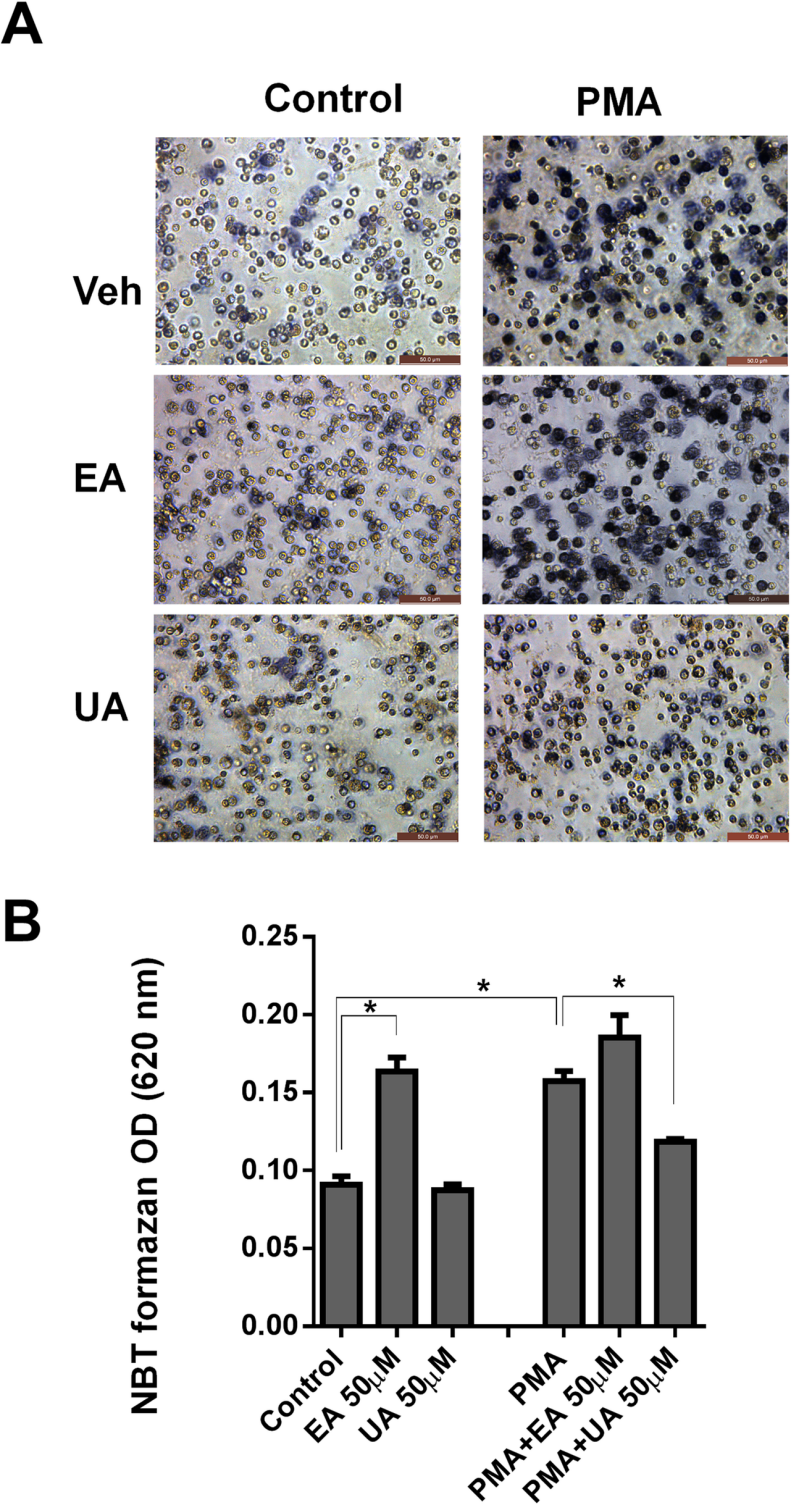
UA attenuated ROS production in PMA-stimulated bone marrow-derived neutrophils (BMDNs). To quantify the superoxide production in BMDN cells, (2x10^5^ /well) were added to a 96 well in triplicates with 1mg/mL NBT in presence or absence of PMA (50 nM) with and without EA or UA (50 μM) and then incubated at 37°C for 3 hrs. NBT assay was performed and absorbance was recorded at 620 nm. (A) Images illustrate formazan production (NBT reduction, blue deposition) in BMDNs at 40X magnification. (B) Bar graphs represent the levels of formazan detected in the NBT reduction assay. Results are expressed as mean ± SEM. Assays were performed in a 96-well plate in triplicates and are representative of three independent experiments. *p < 0.05.

The phorbol myristate acetate (PMA) is a well-known inducer of oxidative stress in neutrophils via induction of ROS production catalyzed by NADPH-oxidase. Hence, we next asked whether UA and EA could inhibit the ROS production in PMA-stimulated neutrophils by employing the nitroblue tetrazolium (NBT) reduction assay to detect the production of intracellular ROS. Accordingly, we observed that UA significantly mitigated PMA-induced NBT formazan production in neutrophils, suggesting that UA could also inhibits ROS production (Fig 6A and 6B). In contrast, EA failed to block ROS production in PMA-treated neutrophils. Intriguingly, EA alone was sufficient to induce ROS production in neutrophils even without PMA stimulation, but this was not the case for UA. We postulated that EA may have pro-oxidant effects in addition to its anti-oxidant properties, which may require further mechanistic study. Nonetheless, our findings herein seem to support the notion that UA may be a superior phytoceutical agent than EA not only in inhibiting MPO and LPO, but also broadly inhibit other pro-oxidative enzymes (i.e. NADPH oxidase).

### UA mediates better protection than EA in mitigating PMA-induced ear edema and MPO activity

**Fig 7:**
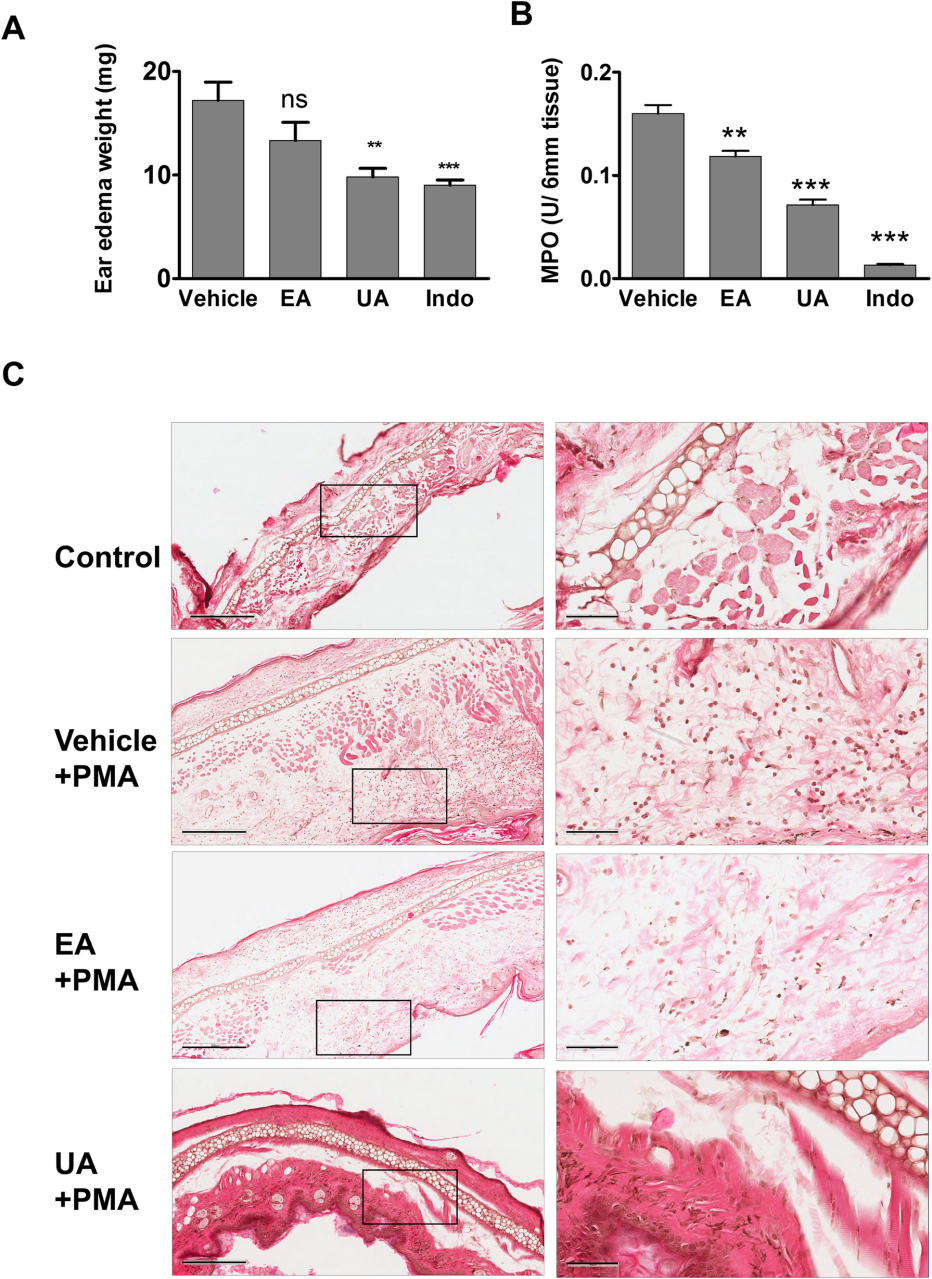
UA treatment reduces PMA-induced ear edema and MPO activity. Mice (n = 5) were orally gavaged with vehicle control (10% glucose), UA (40 mg/kg body weight), EA (40 mg/kg body weight) and indomethacin (10 mg/kg body weight) at 2 hrs prior to applying PMA to right ear and acetone (control) to the left ear. At six hrs post application of PMA, animals were sacrificed by cervical dislocation and (A) weight of edema and (B) MPO activity was evaluated as described in material and method section. Results are expressed as mean ± SEM. *, ** and *** indicates p value < 0.05, 0.01 and 0.001, respectively compared to vehicle group. (C) Representative H&E images of ear edema at 100x magnification (left) and 400x magnification (right) indicate decreased PMA induced inflammation i.e., infiltration of inflammatory cells in UA treated animals compared to vehicle or EA treated animals. The images were captured using Aperio Imagescope and scale bars on 100x and 400x magnifications indicate 300 μm and 60 μm respectively.

To examine the *in vivo* effects of UA and EA, we next tested for their efficacy in treating PMA-induced ear edema in mice. In this acute inflammation model, the severity of disease is reflected by the increased ear edema weight and elevated MPO activity in ear tissue. As shown Fig 7A, mice pre-treated with UA displayed significantly reduced PMA-induced ear edema by 43% when compared to vehicle-treated mice. The extent of protection against PMA-induced ear edema mediated by UA is almost comparable to indomethacin (a positive control) which markedly reduced ear edema by 47.5%. Unlike UA, pretreatment with EA failed to provide significant reduction on PMA-induced ear edema, suggesting that UA display superior efficacy than EA in mitigating acute inflammation. Nevertheless, both UA and EA significantly inhibited the MPO activity induced by PMA, but the inhibition was mediated at a greater extent by UA than EA. Indomethacin also decreased the MPO activity (Fig 7B). Representative H&E images of ear edema further suggest decreased inflammatory cells in UA-treated animals compared to EA treatment or vehicle (Fig 7C). H&E images also suggest that UA treatment also reduced swelling (epidermis thickness) of ear caused by PMA treatment.

## Discussion

Polyphenols-rich fruits such as berries and pomegranate have been extensively investigated for their beneficial effects in the prevention of several human diseases. However, the results across these studies are not consistent, possibly due to the large variation in the gut microbiota and their metabolites which greatly influences their bio-potency. The gut microbial dysbiosis (imbalance in microbial population) especially in gastrointestinal tract-related disorders such as IBD presents yet another major challenge in designing optimal dietary intervention. Several strategies have been sought to improve/alter the gut microbiota through prebiotics, probiotics, antibiotics and fecal microbial transplantations (FMT) to treat patients with IBD [63, 64]. The use probiotics and FMT to treat pouchitis and recurrent *Clostridium difficile* infections were highly successful in most of the cases [65]. Probiotics such as *Bifidobacteria* and *Lactobacillus* have been reported to have some beneficial effects in maintaining remission in IBD patients [66, 67]. We postulate that direct usage of the active metabolites will be beneficial in preventing several chronic disorders and will allow to overcome inter-individual variations in gut microbiota. Bacterial-derived metabolites such as short chain fatty acids (SCFAs), particularly butyrate, have been demonstrated to have beneficial and preventive effects in animal models of autoimmune, IBD and some cancers [68]. Recently, Rusdensky AY’s group demonstrated that metabolites (butyrate) produced by commensal bacteria promote peripheral regulatory T-cell generation [69] and Medzhitov R’s group showed that butyrate regulates intestinal macrophage function via histone deacetylase (HDAC) inhibition [70]. These studies are encouraging to identify novel biological actions, targets and molecular mechanisms of microbial metabolites in preventive and therapeutic settings of several disorders.

Here, we focused on examining one of the microbial metabolite UA for their potency in inhibiting the activity of heme peroxidases (i.e. MPO and LPO). MPO is a hemoprotein expressed in polymorphonuclear neutrophils and macrophages; its production are significantly increased in IBDs [62] and other inflammatory disorders [71]. The release of MPO by activated immune cells promote inflammation, whereas inhibition of MPO can significantly protects from inflammation-mediated tissue damage in IBDs. To the best of our knowledge, there are currently no safe MPO inhibitors that are available to reduce the severity of MPO-driven inflammation. Therefore, the use of naturally available dietary and microbial metabolites to modulate MPO activity present an attractive approach in treating inflammatory disorders. In this study, we demonstrate that physiological concentrations of UA significantly inhibited the activity of MPO and LPO. It is pertinent to highlight that microbial-derived UA is a more potent inhibitor than its parent compound EA, indicating collegial nature of microbiota and its metabolites to protect from increased inflammation. EA contains catechol group (2 OH groups adjacent on phenyl group), which are capable of chelating iron. Interestingly, gut microbiota metabolizes EA by series of enzymatic reactions and removes these catechol groups from EA to generate UA. Such modification provides several notable advantage to the gut microbiota: (1) it removes iron-binding EA from competing with bacterial iron acquisition, and (2) augments the inhibitory activity of UA which, unlike EA, could not be suppressed by iron-binding.

Iron deficiency anaemia is one of major problem that is commonly associated with IBD. Indeed, one third of IBD patients were reported to suffer from recurrent anaemia. Unfortunately, oral iron treatment is limited by poor absorption, intolerance, and induction of oxidative stress at the site of bowel inflammation. Therefore, presence of bacteria that is responsible (currently unknown) for production UA from EA is beneficial and potentially lead to increase free iron and decrease in anaemic state in colonic disorders, including IBDs. In this study, we demonstrated that UA lacked the ability to chelate iron, unlike EA and EGCG that display iron-binding properties. We envision that the therapeutic use of EA may not be ideal since its iron-chelating property may further aggravate iron deficiency anaemia associated with IBD. In this regard, we postulate that UA may be a better therapeutic metabolite than EA, given the superior peroxidase inhibitory activity and non-iron binding properties of UA.

The potency of UA in inhibiting the activity of heme peroxidase prompted us to investigate the potential underlying mechanisms that are involved. In this study, we performed spectral analysis using LPO as our model heme peroxidase; however, we could not employ MPO in our spectral analysis due to technical limitation (i.e. amount supplied is inadequate). Heme peroxidase (e.g. LPO) displays absorbance spectra with λmax at 412 nm due to the presence of its heme moiety group. The addition of H_2_O_2_ to LPO would result in the formation of the peroxidase radical intermediate oxoiron with λmax at 430 nm. Interestingly, the treatment with UA or EA prematurely inactivates the peroxidase-catalyzed reaction and reversed the oxoiron group to its normal native state. The inactivation of peroxidase activity by UA and EA suggests that these compounds are capable of negating the production of hypohalous acid by LPO and potentially MPO as well. This outcome could be possibly be attributed to the donation of electrons by UA to the oxoiron state of the heme and thus, revert to its native state. The UA-mediated inhibition of MPO may be beneficial in mitigating hypohalous acid-mediated tissue damage in the intestines during increased inflammatory conditions.

Lipocalin (Lcn2) and its human ortholog NGAL sequester iron-containing siderophore as an innate immune mechanism to limit the growth of bacteria, especially during infection or inflammation. Accordingly, the mice lacking Lcn2 are more susceptible to bacterial infection compare to wild type mice [72]. Herein, we observed that 10 μM of UA inhibits approximately 40% of MPO activity. Interestingly, in the presence of Lcn2/NGAL, the inhibitory activity of UA is significantly mitigated (less than 10% inhibition of MPO), suggesting that UA can be sequestered by Lcn2 or NGAL. The findings that Lcn2/NGAL preserves the activity of MPO from being inactivated by UA are reminiscent of our previous studies with EGCG and bacterial enterobactin (Ent) [51, 60]. Similar to UA, EGCG and Ent are potent MPO inhibitors that also loses their efficacy upon complexing with Lcn2/NGAL [51, 60]. It is possible that Lcn2/NGAL system is evolutionarily designed to selectively remove the iron-binding siderophore and polyphenols so that immune system can clear the bacterial infections. Future studies on UA-based therapeutics may need to consider the counter-regulatory function of Lcn2/NGAL when optimizing the doses UA to be given to IBD patients.

PMNs produce a variety of reactive oxygen species (ROS), including superoxide anion (O_2_^•−^), hydrogen peroxide (H_2_O_2_), hydroxyl radical (^•^OH), and hypochlorous acid (HOCl) upon stimulation by soluble agonists, such as phorbol ester, chemotactic peptides, and calcium ionophore. The phagocyte activation leads to increase in the production of superoxide anion leading to oxidative burst. In general, stimulation of neutrophils by PMA increases the ROS formation, which can be quantified by NBT assay. In this study, we further demonstrated that UA treatment could mitigate PMA-induced ROS formation in neutrophils (*in vitro*), but such protective effect was not observed with EA. Instead, we found that EA alone could induce ROS formation, suggesting that EA, but not UA, may have pro-oxidant properties that need to be further investigated. Furthermore, we confirmed these observations in *in vivo* model, where oral treatment of UA significantly reduced PMA induced ear edema and MPO activity suggesting its potential role as an anti-inflammatory agent. In support of these observations including our unpublished data, it was demonstrated that UA is able to markedly reduce carrageen-induced paw edema as well as LPS mediated anti-inflammatory activities in other models [73].

In summary, our studies demonstrate that UA is a potent inhibitor of MPO compared to its parent compound, EA. The inability of UA to bind iron allows it to retain its peroxidase-inhibitory activity in the presence of ferric iron, whereas EA loses its bioactivity significantly upon binding to iron. However, the innate immune protein NGAL could reverses the UA-mediated inhibition of heme peroxidase activity, implicating its regulation by host-defense and inflammatory activities. Finally, we have demonstrated UA potent inhibitory activities on PMA induced ROS production as well as significant reduction of PMA-induced ear edema in a mouse model. These studies form strong evidence that gut microbiota-derived active metabolites are critical in controlling inflammatory pathways.

## Supporting Information

S1 Scheme Schematic diagram of Urolithin A (UA) synthesis. (TIF)

S1 Fig ^1^H-NMR spectra of Urolithin A (UA). ^**1**^**H-NMR** DMSO-d6: 800MHz: δ: 10.22 (1H, s), 10.15 (1H, s), 8.09–8.07 (1H, d, J = 12), 8.00–7.99 (1H, d, J = 12), 7.50 (1H, s), 7.31–7.30 (1H, m), 6.80–6.78 (1H, m), 6.72 (1H, s).
(TIF)

S2 Fig ^13^C-NMR spectra of Urolithin A (UA). ^**13**^**C-NMR:** DMSO-d6: 800MHz: δ: 160.60, 158.53, 156.93, 150.88, 126.94, 124.14, 123.77, 123.54, 120.15, 113.52, 113.02, 109.82, 102.83.
(TIF)

S3 Fig HPLC spectra of Urolithin A (UA). (TIF)

S4 Fig Potential EA binding mode to iron. (TIF)

## References

[1] O'Hara AM, Shanahan F. The gut flora as a forgotten organ. EMBO Rep. 2006;7(7):688–93. 10.1038/sj.embor.7400731 16819463 PMC1500832

[2] Marchesi JR, Adams DH, Fava F, Hermes GD, Hirschfield GM, Hold G, et al The gut microbiota and host health: a new clinical frontier. Gut. 2016;65(2):330–9. 10.1136/gutjnl-2015-309990 26338727 PMC4752653

[3] Flint HJ, Scott KP, Louis P, Duncan SH. The role of the gut microbiota in nutrition and health. Nature reviews Gastroenterology & hepatology. 2012;9(10):577–89. 10.1038/nrgastro.2012.156 . 22945443

[4] Sekirov I, Russell SL, Antunes LC, Finlay BB. Gut microbiota in health and disease. Physiol Rev. 2010;90(3):859–904. 10.1152/physrev.00045.2009 . 20664075

[5] Rutledge PJ, Challis GL. Discovery of microbial natural products by activation of silent biosynthetic gene clusters. Nat Rev Microbiol. 2015;13(8):509–23. 10.1038/nrmicro3496 . 26119570

[6] Tomkovich S, Jobin C. Microbiota and host immune responses: a love-hate relationship. Immunology. 2016;147(1):1–10. 10.1111/imm.12538 26439191 PMC4693877

[7] Belkaid Y, Hand TW. Role of the microbiota in immunity and inflammation. Cell. 2014;157(1):121–41. 10.1016/j.cell.2014.03.011 24679531 PMC4056765

[8] Belkaid Y, Naik S. Compartmentalized and systemic control of tissue immunity by commensals. Nature immunology. 2013;14(7):646–53. 10.1038/ni.2604 23778791 PMC3845005

[9] Sobhani I, Tap J, Roudot-Thoraval F, Roperch JP, Letulle S, Langella P, et al Microbial dysbiosis in colorectal cancer (CRC) patients. PloS one. 2011;6(1):e16393 10.1371/journal.pone.0016393 21297998 PMC3029306

[10] Schwabe RF, Jobin C. The microbiome and cancer. Nature reviews Cancer. 2013;13(11):800–12. 10.1038/nrc3610 . 24132111 PMC3986062

[11] Kinross JM, Darzi AW, Nicholson JK. Gut microbiome-host interactions in health and disease. Genome Med. 2011;3(3):14 Epub 2011/03/12. 10.1186/gm228 . 21392406 PMC3092099

[12] Chung H, Kasper DL. Microbiota-stimulated immune mechanisms to maintain gut homeostasis. Curr Opin Immunol. 2010;22(4):455–60. Epub 2010/07/27. 10.1016/j.coi.2010.06.008 . 20656465

[13] Lee YK, Mazmanian SK. Has the microbiota played a critical role in the evolution of the adaptive immune system? Science. 2010;330(6012):1768–73. Epub 2011/01/06. 10.1126/science.1195568 . 21205662 PMC3159383

[14] Kuczynski J, Costello EK, Nemergut DR, Zaneveld J, Lauber CL, Knights D, et al Direct sequencing of the human microbiome readily reveals community differences. Genome Biol. 2010;11(5):210 Epub 2010/05/06. 10.1186/gb-2010-11-5-210 20441597 PMC2898070

[15] Arthur JC, Jobin C. The struggle within: microbial influences on colorectal cancer. Inflamm Bowel Dis. 2011;17(1):396–409. Epub 2010/09/18. 10.1002/ibd.21354 . 20848537 PMC3376405

[16] Candela M, Guidotti M, Fabbri A, Brigidi P, Franceschi C, Fiorentini C. Human intestinal microbiota: cross-talk with the host and its potential role in colorectal cancer. Crit Rev Microbiol. 2011;37(1):1–14. Epub 2010/09/30. 10.3109/1040841X.2010.501760 . 20874522

[17] Ley RE, Hamady M, Lozupone C, Turnbaugh PJ, Ramey RR, Bircher JS, et al Evolution of mammals and their gut microbes. Science. 2008;320(5883):1647–51. Epub 2008/05/24. 10.1126/science.1155725 18497261 PMC2649005

[18] Turnbaugh PJ, Ley RE, Mahowald MA, Magrini V, Mardis ER, Gordon JI. An obesity-associated gut microbiome with increased capacity for energy harvest. Nature. 2006;444(7122):1027–31. Epub 2006/12/22. 10.1038/nature05414 . 17183312

[19] Uronis JM, Muhlbauer M, Herfarth HH, Rubinas TC, Jones GS, Jobin C. Modulation of the intestinal microbiota alters colitis-associated colorectal cancer susceptibility. PloS one. 2009;4(6):e6026 Epub 2009/06/25. 10.1371/journal.pone.0006026 19551144 PMC2696084

[20] Tlaskalova-Hogenova H, Stepankova R, Kozakova H, Hudcovic T, Vannucci L, Tuckova L, et al The role of gut microbiota (commensal bacteria) and the mucosal barrier in the pathogenesis of inflammatory and autoimmune diseases and cancer: contribution of germ-free and gnotobiotic animal models of human diseases. Cell Mol Immunol. 2011;8(2):110–20. Epub 2011/02/01. 10.1038/cmi.2010.67 . 21278760 PMC4003137

[21] Mukherji A, Kobiita A, Ye T, Chambon P. Homeostasis in intestinal epithelium is orchestrated by the circadian clock and microbiota cues transduced by TLRs. Cell. 2013;153(4):812–27. 10.1016/j.cell.2013.04.020 . 23663780

[22] Alasalvar C, Bolling BW. Review of nut phytochemicals, fat-soluble bioactives, antioxidant components and health effects. The British journal of nutrition. 2015;113 Suppl 2:S68–78. 10.1017/S0007114514003729 . 26148924

[23] Zhang HM, Zhao L, Li H, Xu H, Chen WW, Tao L. Research progress on the anticarcinogenic actions and mechanisms of ellagic acid. Cancer Biol Med. 2014;11(2):92–100. 10.7497/j.issn.2095-3941.2014.02.004 25009751 PMC4069806

[24] Heber D. Multitargeted therapy of cancer by ellagitannins. Cancer letters. 2008;269(2):262–8. 10.1016/j.canlet.2008.03.043 . 18468784

[25] Girish C, Pradhan SC. Drug development for liver diseases: focus on picroliv, ellagic acid and curcumin. Fundam Clin Pharmacol. 2008;22(6):623–32. 10.1111/j.1472-8206.2008.00618.x . 19049667

[26] Jurenka JS. Therapeutic applications of pomegranate (Punica granatum L.): a review. Altern Med Rev. 2008;13(2):128–44. . 18590349

[27] Bell C, Hawthorne S. Ellagic acid, pomegranate and prostate cancer—a mini review. The Journal of pharmacy and pharmacology. 2008;60(2):139–44. 10.1211/jpp.60.2.0001 . 18237460

[28] Banihani S, Swedan S, Alguraan Z. Pomegranate and type 2 diabetes. Nutr Res. 2013;33(5):341–8. 10.1016/j.nutres.2013.03.003 . 23684435

[29] Xu KZ, Zhu C, Kim MS, Yamahara J, Li Y. Pomegranate flower ameliorates fatty liver in an animal model of type 2 diabetes and obesity. Journal of ethnopharmacology. 2009;123(2):280–7. 10.1016/j.jep.2009.03.035 . 19429373

[30] McFarlin BK, Strohacker KA, Kueht ML. Pomegranate seed oil consumption during a period of high-fat feeding reduces weight gain and reduces type 2 diabetes risk in CD-1 mice. The British journal of nutrition. 2009;102(1):54–9. 10.1017/S0007114508159001 . 19079947

[31] Espin JC, Larrosa M, Garcia-Conesa MT, Tomas-Barberan F. Biological significance of urolithins, the gut microbial ellagic Acid-derived metabolites: the evidence so far. Evidence-based complementary and alternative medicine: eCAM. 2013;2013:270418 10.1155/2013/270418 23781257 PMC3679724

[32] Cerda B, Llorach R, Ceron JJ, Espin JC, Tomas-Barberan FA. Evaluation of the bioavailability and metabolism in the rat of punicalagin, an antioxidant polyphenol from pomegranate juice. European journal of nutrition. 2003;42(1):18–28. 10.1007/s00394-003-0396-4 . 12594538

[33] Cerda B, Espin JC, Parra S, Martinez P, Tomas-Barberan FA. The potent in vitro antioxidant ellagitannins from pomegranate juice are metabolised into bioavailable but poor antioxidant hydroxy-6H-dibenzopyran-6-one derivatives by the colonic microflora of healthy humans. European journal of nutrition. 2004;43(4):205–20. 10.1007/s00394-004-0461-7 . 15309440

[34] Mertens-Talcott SU, Jilma-Stohlawetz P, Rios J, Hingorani L, Derendorf H. Absorption, metabolism, and antioxidant effects of pomegranate (Punica granatum l.) polyphenols after ingestion of a standardized extract in healthy human volunteers. Journal of agricultural and food chemistry. 2006;54(23):8956–61. 10.1021/jf061674h . 17090147

[35] González-Sarrías A, García-Villalba R, Núñez-Sánchez MÁ, Tomé-Carneiro J, Zafrilla P, Mulero J, et al Identifying the limits for ellagic acid bioavailability: A crossover pharmacokinetic study in healthy volunteers after consumption of pomegranate extracts. Journal of Functional Foods. 2015;19, Part A:225–35. 10.1016/j.jff.2015.09.019.

[36] Cerda B, Periago P, Espin JC, Tomas-Barberan FA. Identification of urolithin a as a metabolite produced by human colon microflora from ellagic acid and related compounds. Journal of agricultural and food chemistry. 2005;53(14):5571–6. 10.1021/jf050384i . 15998116

[37] Selma MV, Beltran D, Garcia-Villalba R, Espin JC, Tomas-Barberan FA. Description of urolithin production capacity from ellagic acid of two human intestinal Gordonibacter species. Food & function. 2014;5(8):1779–84. 10.1039/c4fo00092g . 24909569

[38] Larrosa M, Gonzalez-Sarrias A, Yanez-Gascon MJ, Selma MV, Azorin-Ortuno M, Toti S, et al Anti-inflammatory properties of a pomegranate extract and its metabolite urolithin-A in a colitis rat model and the effect of colon inflammation on phenolic metabolism. J Nutr Biochem. 2010;21(8):717–25. 10.1016/j.jnutbio.2009.04.012 . 19616930

[39] Espin JC, Gonzalez-Barrio R, Cerda B, Lopez-Bote C, Rey AI, Tomas-Barberan FA. Iberian pig as a model to clarify obscure points in the bioavailability and metabolism of ellagitannins in humans. J Agric Food Chem. 2007;55(25):10476–85. 10.1021/jf0723864 . 17990850

[40] Gonzalez-Sarrias A, Gimenez-Bastida JA, Garcia-Conesa MT, Gomez-Sanchez MB, Garcia-Talavera NV, Gil-Izquierdo A, et al Occurrence of urolithins, gut microbiota ellagic acid metabolites and proliferation markers expression response in the human prostate gland upon consumption of walnuts and pomegranate juice. Mol Nutr Food Res. 2010;54(3):311–22. 10.1002/mnfr.200900152 . 19885850

[41] Seeram NP, Aronson WJ, Zhang Y, Henning SM, Moro A, Lee RP, et al Pomegranate ellagitannin-derived metabolites inhibit prostate cancer growth and localize to the mouse prostate gland. Journal of agricultural and food chemistry. 2007;55(19):7732–7. 10.1021/jf071303g . 17722872

[42] Odobasic D, Kitching AR, Holdsworth SR. Neutrophil-Mediated Regulation of Innate and Adaptive Immunity: The Role of Myeloperoxidase. J Immunol Res. 2016;2016:2349817 10.1155/2016/2349817 26904693 PMC4745373

[43] Carpena X, Vidossich P, Schroettner K, Calisto BM, Banerjee S, Stampler J, et al Essential role of proximal histidine-asparagine interaction in mammalian peroxidases. The Journal of biological chemistry. 2009;284(38):25929–37. 10.1074/jbc.M109.002154 19608745 PMC2757993

[44] Heinecke JW, Li W, Francis GA, Goldstein JA. Tyrosyl radical generated by myeloperoxidase catalyzes the oxidative cross-linking of proteins. The Journal of clinical investigation. 1993;91(6):2866–72. 10.1172/JCI116531 8390491 PMC443356

[45] Hampton MB, Kettle AJ, Winterbourn CC. Inside the neutrophil phagosome: oxidants, myeloperoxidase, and bacterial killing. Blood. 1998;92(9):3007–17. . 9787133

[46] Davies MJ. Myeloperoxidase-derived oxidation: mechanisms of biological damage and its prevention. Journal of clinical biochemistry and nutrition. 2011;48(1):8–19. 10.3164/jcbn.11-006FR 21297906 PMC3022070

[47] Klebanoff SJ. Myeloperoxidase: friend and foe. Journal of leukocyte biology. 2005;77(5):598–625. 10.1189/jlb.1204697 . 15689384

[48] Davies MJ, Hawkins CL, Pattison DI, Rees MD. Mammalian heme peroxidases: from molecular mechanisms to health implications. Antioxidants & redox signaling. 2008;10(7):1199–234. 10.1089/ars.2007.1927 . 18331199

[49] Rees MD, Kennett EC, Whitelock JM, Davies MJ. Oxidative damage to extracellular matrix and its role in human pathologies. Free radical biology & medicine. 2008;44(12):1973–2001. 10.1016/j.freeradbiomed.2008.03.016 . 18423414

[50] Bensalem S, Soubhye J, Aldib I, Bournine L, Nguyen AT, Vanhaeverbeek M, et al Inhibition of myeloperoxidase activity by the alkaloids of Peganum harmala L. (Zygophyllaceae). Journal of ethnopharmacology. 2014;154(2):361–9. 10.1016/j.jep.2014.03.070 . 24746482

[51] Yeoh BS, Aguilera Olvera R, Singh V, Xiao X, Kennett MJ, Joe B, et al Epigallocatechin-3-Gallate Inhibition of Myeloperoxidase and Its Counter-Regulation by Dietary Iron and Lipocalin 2 in Murine Model of Gut Inflammation. The American journal of pathology. 2016;186(4):912–26. 10.1016/j.ajpath.2015.12.004 . 26968114 PMC5848242

[52] Capeillere-Blandin C. Oxidation of guaiacol by myeloperoxidase: a two-electron-oxidized guaiacol transient species as a mediator of NADPH oxidation. The Biochemical journal. 1998;336 (Pt 2):395–404. 9820817 10.1042/bj3360395 PMC1219884

[53] Schwyn B, Neilands JB. Universal chemical assay for the detection and determination of siderophores. Analytical biochemistry. 1987;160(1):47–56. . 2952030 10.1016/0003-2697(87)90612-9

[54] Payne SM. Detection, isolation, and characterization of siderophores. Methods in enzymology. 1994;235:329–44. . 8057905 10.1016/0076-6879(94)35151-1

[55] Swamydas M, Lionakis MS. Isolation, purification and labeling of mouse bone marrow neutrophils for functional studies and adoptive transfer experiments. J Vis Exp. 2013;(77):e50586. 10.3791/50586 23892876 PMC3732092

[56] Choi HS, Kim JW, Cha YN, Kim C. A quantitative nitroblue tetrazolium assay for determining intracellular superoxide anion production in phagocytic cells. J Immunoassay Immunochem. 2006;27(1):31–44. 10.1080/15321810500403722 . 16450867

[57] Griswold DE, Martin LD, Badger AM, Breton J, Chabot-Fletcher M. Evaluation of the cutaneous anti-inflammatory activity of azaspiranes. Inflamm Res. 1998;47(2):56–61. . 9535542 10.1007/s000110050270

[58] Bradley PP, Priebat DA, Christensen RD, Rothstein G. Measurement of cutaneous inflammation: estimation of neutrophil content with an enzyme marker. J Invest Dermatol. 1982;78(3):206–9. . 6276474 10.1111/1523-1747.ep12506462

[59] Perron NR, Brumaghim JL. A review of the antioxidant mechanisms of polyphenol compounds related to iron binding. Cell Biochem Biophys. 2009;53(2):75–100. 10.1007/s12013-009-9043-x . 19184542

[60] Singh V, Yeoh BS, Xiao X, Kumar M, Bachman M, Borregaard N, et al Interplay between enterobactin, myeloperoxidase and lipocalin 2 regulates E. coli survival in the inflamed gut. Nature communications. 2015;6:7113 10.1038/ncomms8113 . 25964185 PMC6336494

[61] Oikonomou KA, Kapsoritakis AN, Theodoridou C, Karangelis D, Germenis A, Stefanidis I, et al Neutrophil gelatinase-associated lipocalin (NGAL) in inflammatory bowel disease: association with pathophysiology of inflammation, established markers, and disease activity. Journal of gastroenterology. 2012;47(5):519–30. 10.1007/s00535-011-0516-5 . 22200942

[62] Feng BS, Chen X, Li P, Zheng PY, Chong J, Cho DB, et al Expression of integrin alphavbeta6 in the intestinal epithelial cells of patients with inflammatory bowel disease. N Am J Med Sci. 2009;1(4):200–4. 10.4297/najms.2009.4200 22666696 PMC3364666

[63] Guinane CM, Cotter PD. Role of the gut microbiota in health and chronic gastrointestinal disease: understanding a hidden metabolic organ. Therap Adv Gastroenterol. 2013;6(4):295–308. 10.1177/1756283X13482996 23814609 PMC3667473

[64] Vyas U, Ranganathan N. Probiotics, prebiotics, and synbiotics: gut and beyond. Gastroenterol Res Pract. 2012;2012:872716 10.1155/2012/872716 23049548 PMC3459241

[65] Borody TJ, Khoruts A. Fecal microbiota transplantation and emerging applications. Nature reviews Gastroenterology & hepatology. 2012;9(2):88–96. 10.1038/nrgastro.2011.244 . 22183182

[66] Andreasen AS, Larsen N, Pedersen-Skovsgaard T, Berg RM, Moller K, Svendsen KD, et al Effects of Lactobacillus acidophilus NCFM on insulin sensitivity and the systemic inflammatory response in human subjects. The British journal of nutrition. 2010;104(12):1831–8. 10.1017/S0007114510002874 . 20815975

[67] Kadooka Y, Sato M, Imaizumi K, Ogawa A, Ikuyama K, Akai Y, et al Regulation of abdominal adiposity by probiotics (Lactobacillus gasseri SBT2055) in adults with obese tendencies in a randomized controlled trial. Eur J Clin Nutr. 2010;64(6):636–43. 10.1038/ejcn.2010.19 . 20216555

[68] Canani RB, Costanzo MD, Leone L, Pedata M, Meli R, Calignano A. Potential beneficial effects of butyrate in intestinal and extraintestinal diseases. World journal of gastroenterology: WJG. 2011;17(12):1519–28. 10.3748/wjg.v17.i12.1519 21472114 PMC3070119

[69] Arpaia N, Campbell C, Fan X, Dikiy S, van der Veeken J, deRoos P, et al Metabolites produced by commensal bacteria promote peripheral regulatory T-cell generation. Nature. 2013;504(7480):451–5. 10.1038/nature12726 24226773 PMC3869884

[70] Chang PV, Hao L, Offermanns S, Medzhitov R. The microbial metabolite butyrate regulates intestinal macrophage function via histone deacetylase inhibition. Proceedings of the National Academy of Sciences of the United States of America. 2014;111(6):2247–52. 10.1073/pnas.1322269111 24390544 PMC3926023

[71] Podrez EA, Abu-Soud HM, Hazen SL. Myeloperoxidase-generated oxidants and atherosclerosis. Free radical biology & medicine. 2000;28(12):1717–25. . 10946213 10.1016/s0891-5849(00)00229-x

[72] Flo TH, Smith KD, Sato S, Rodriguez DJ, Holmes MA, Strong RK, et al Lipocalin 2 mediates an innate immune response to bacterial infection by sequestrating iron. Nature. 2004;432(7019):917–21. 10.1038/nature03104 . 15531878

[73] Ishimoto H, Shibata M, Myojin Y, Ito H, Sugimoto Y, Tai A, et al In vivo anti-inflammatory and antioxidant properties of ellagitannin metabolite urolithin A. Bioorg Med Chem Lett. 2011;21(19):5901–4. 10.1016/j.bmcl.2011.07.086 . 21843938

