# Supplementary figures and images for: Gut Microbiota Conversion of Dietary Ellagic Acid into Bioactive Phytoceutical Urolithin A Inhibits Heme Peroxidases
Source: PLoS One. 2016 Jun 2;11(6):e0156811. doi: 10.1371/journal.pone.0156811 (PMC4890745; doi:10.1371/journal.pone.0156811)

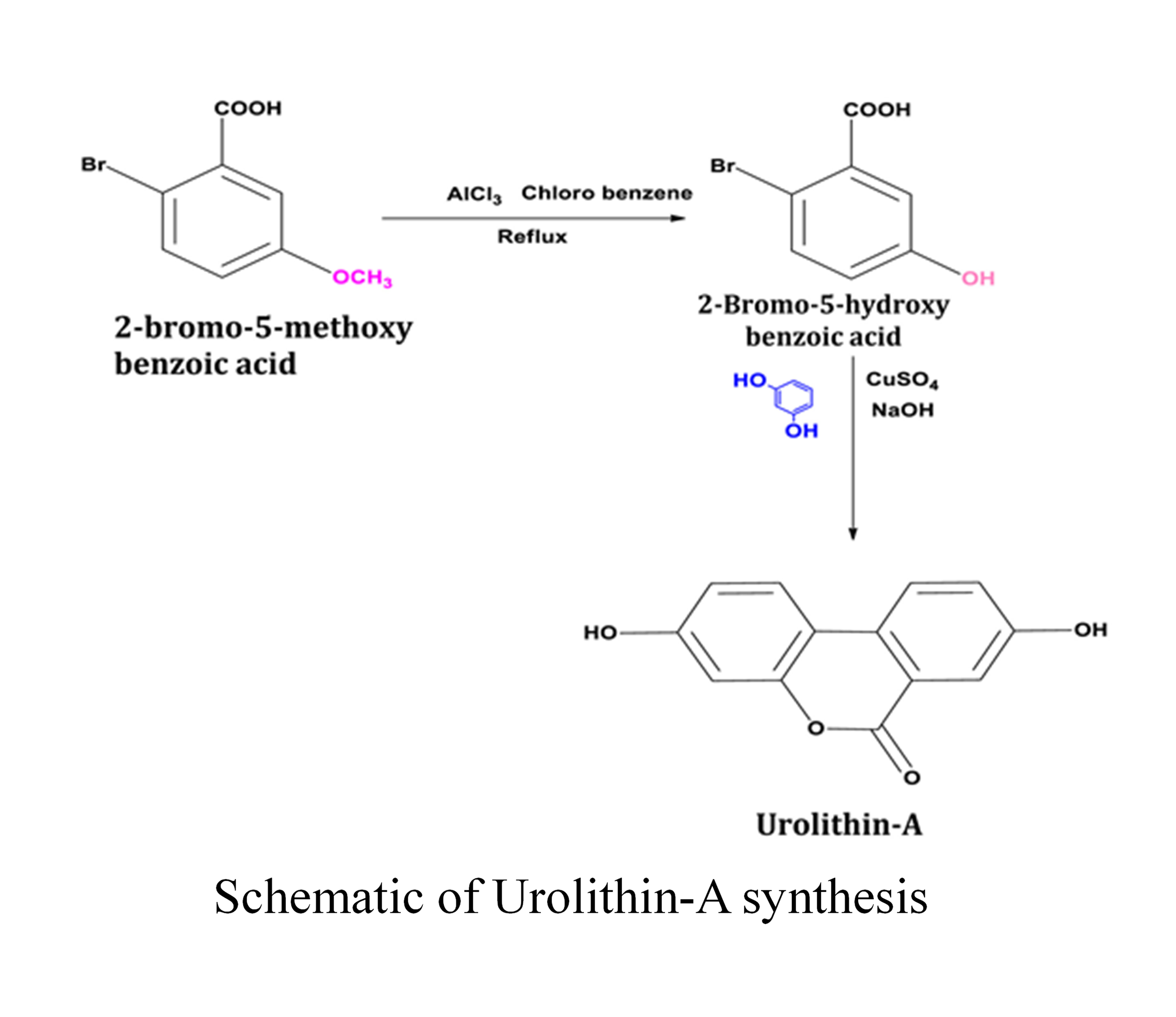

Supplement: S1 Scheme — (TIF) [file pone.0156811.s001.tif]

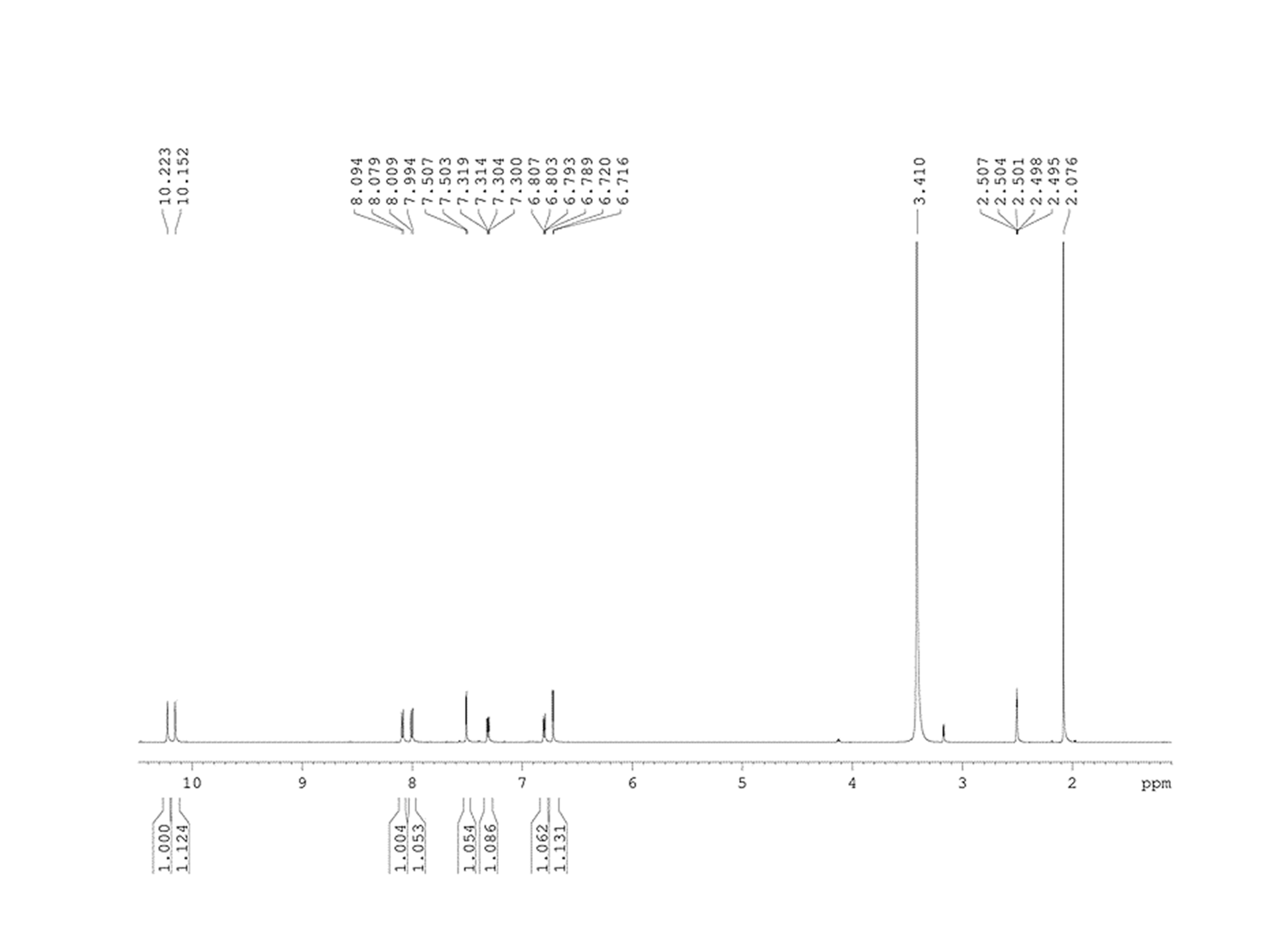

Supplement: S1 Fig — 1H-NMR DMSO-d6: 800MHz: δ: 10.22 (1H, s), 10.15 (1H, s), 8.09–8.07 (1H, d, J = 12), 8.00–7.99 (1H, d, J = 12), 7.50 (1H, s), 7.31–7.30 (1H, m), 6.80–6.78 (1H, m), 6.72 (1H, s). (TIF) [file pone.0156811.s002.tif]

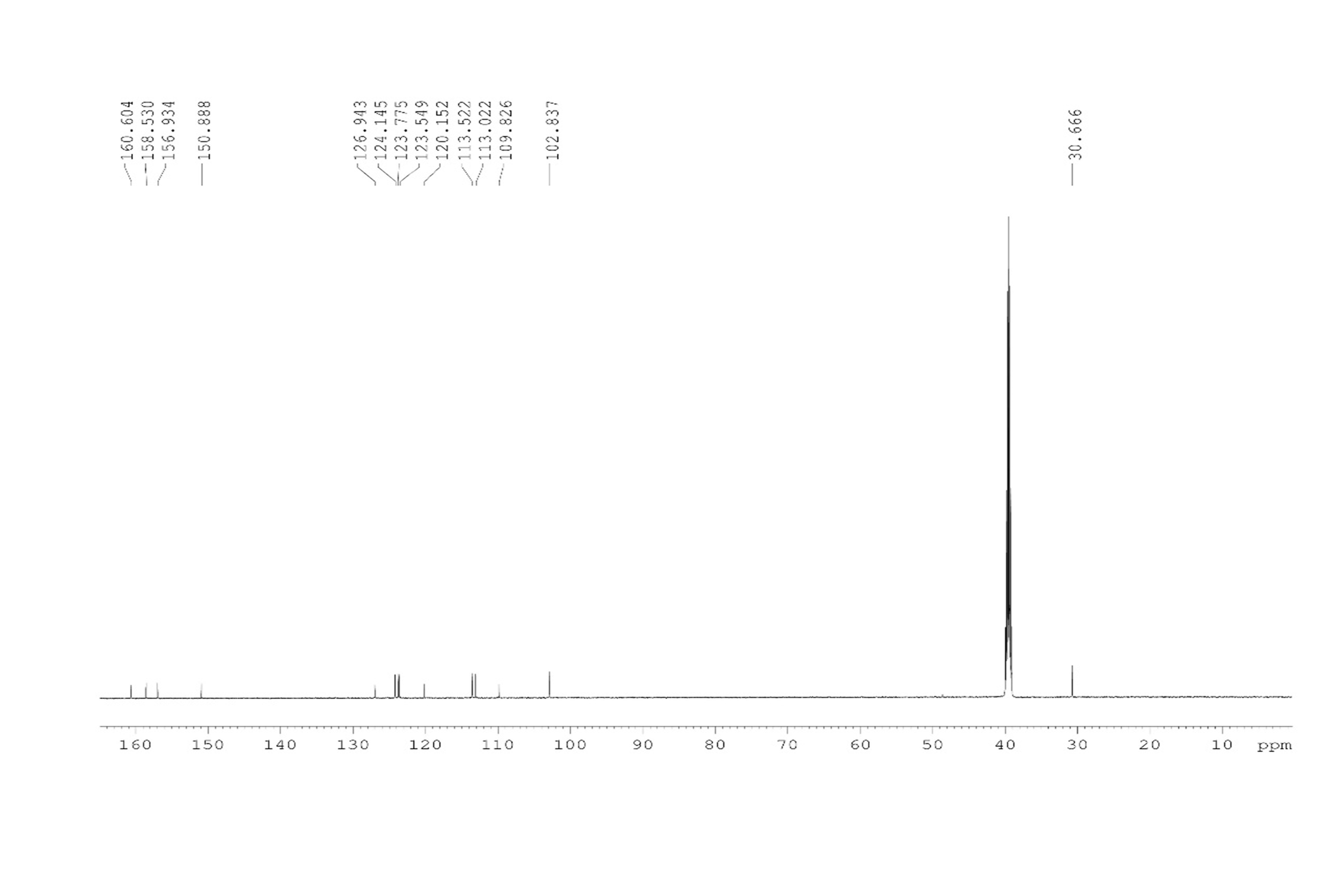

Supplement: S2 Fig — 13C-NMR: DMSO-d6: 800MHz: δ: 160.60, 158.53, 156.93, 150.88, 126.94, 124.14, 123.77, 123.54, 120.15, 113.52, 113.02, 109.82, 102.83. (TIF) [file pone.0156811.s003.tif]

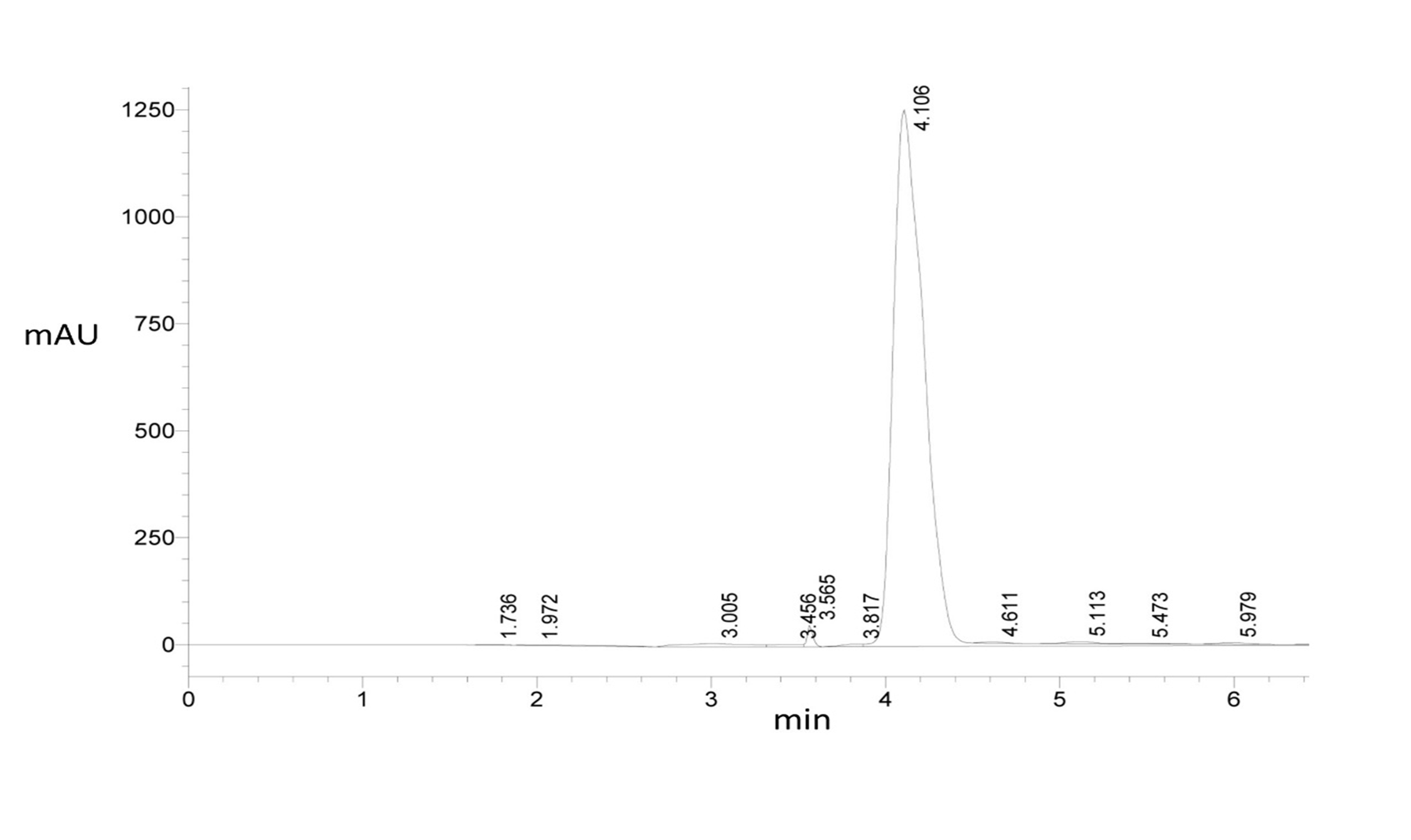

Supplement: S3 Fig — (TIF) [file pone.0156811.s004.tif]

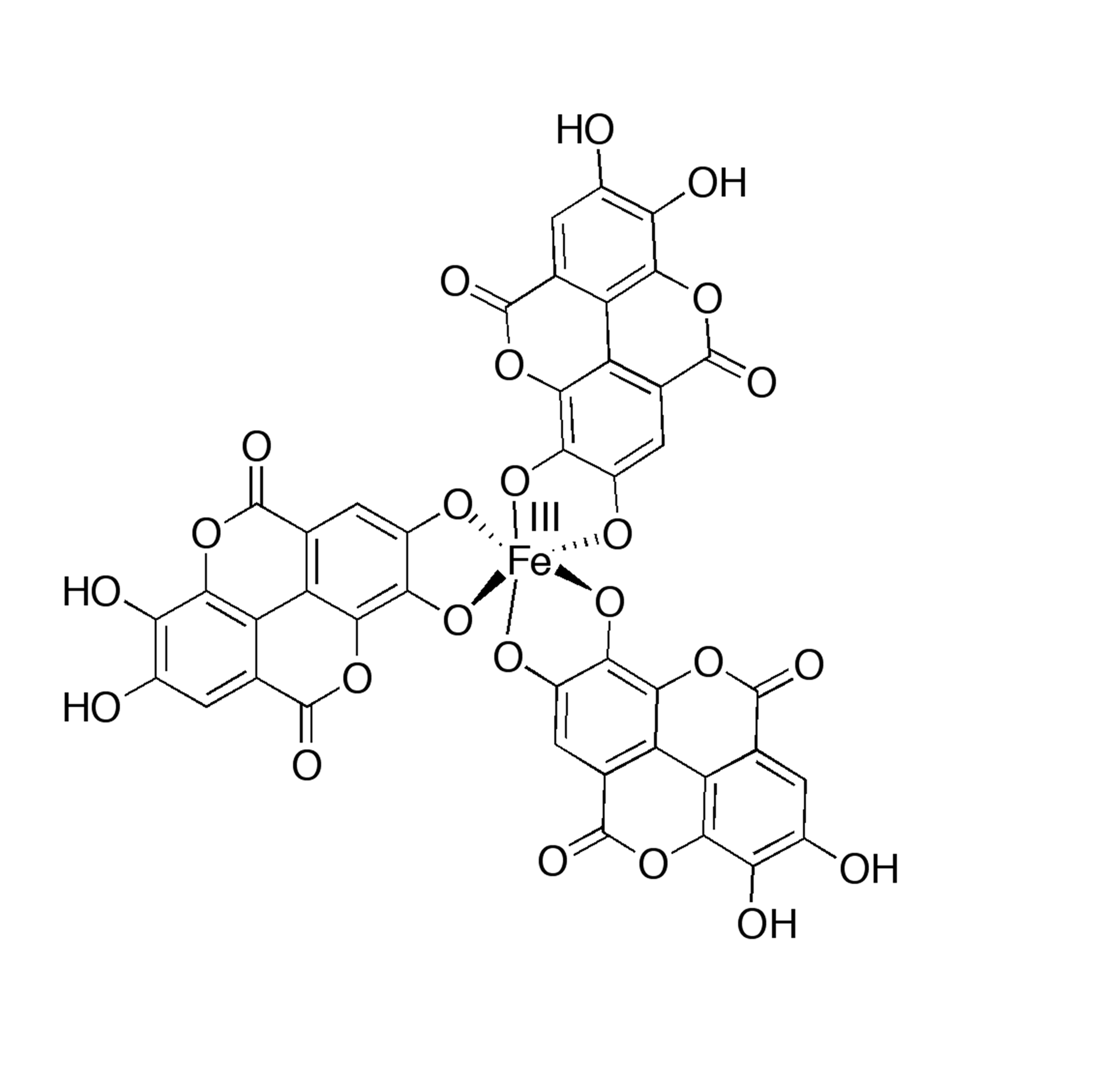

Supplement: S4 Fig — (TIF) [file pone.0156811.s005.tif]
